# Intraperitoneal paclitaxel combined with FOLFOX/CAPOX plus bevacizumab for colorectal cancer with peritoneal carcinomatosis (the iPac-02 trial): study protocol of a single arm, multicenter, phase 2 study

**DOI:** 10.1007/s00384-023-04434-5

**Published:** 2023-06-20

**Authors:** Koji Murono, Yuichiro Yokoyama, Hiroaki Nozawa, Kazuhito Sasaki, Shigenobu Emoto, Hiroyuki Matsuzaki, Kosuke Kashiwabara, Hironori Ishigami, Yoshimasa Gohda, Hironori Yamaguchi, Joji Kitayama, Soichiro Ishihara

**Affiliations:** 1 Division of Surgical Oncology, Department of Surgery, Faculty of Medicine, the University of Tokyo, 7-3-1 Hongo, Bunkyo-ku, Tokyo, 113-8655 Japan; 2 Interfaculty Initiative in Information Studies, Department of Biostatistics, School of Public Health, Graduate School of Medicine, The University of Tokyo, Tokyo, Japan; 3 Department of Chemotherapy, The University of Tokyo Hospital, Tokyo, Japan; 4 Department of Gastroenterology and Hepatology, National Center for Global Health and Medicine, Tokyo, Japan; 5 Department of Clinical Oncology, Jichi Medical University Hospital, Shimotsuke, Japan; 6 Clinical Research Center, Department of Surgery, Jichi Medical University, Shimotsuke, Japan

**Keywords:** Phase II trial, Intraperitoneal paclitaxel, Colorectal cancer, Peritoneal carcinomatosis

## Abstract

**Background:**

The safety of intraperitoneally administrated paclitaxel (op PTX) was demonstrated in the phase I trial of ip PTX combined with conventional systemic chemotherapy for colorectal cancer with peritoneal carcinomatosis. Moreover, the median survival time was 29.3 months, which was longer than that observed in previous studies. Here, we planned the phase II trial of ip PTX: the iPac-02 trial.

**Methods:**

This multicenter, open-label, single assignment interventional clinical study includes patients with colorectal cancer with unresectable peritoneal carcinomatosis. FOLFOX-bevacizumab or CAPOX-bevacizumab is administered concomitantly as systemic chemotherapy. PTX 20 mg/m^2^ is administered weekly through the peritoneal access port in addition to these conventional systemic chemotherapies. The response rate is the primary endpoint. Progression-free survival, overall survival, peritoneal cancer index improvement rate, rate of negative peritoneal lavage cytology, safety, and response rate to peritoneal metastases are the secondary endpoints. A total of 38 patients are included in the study. In the interim analysis, the study will continue to the second stage if at least 4 of the first 14 patients respond to the study treatment. The study has been registered at the Japan Registry of Clinical Trials (jRCT2031220110).

**Results:**

We previously conducted phase I trial of ip PTX combined with conventional systemic chemotherapy for colorectal cancer with peritoneal carcinomatosis [1]. In the study, three patients underwent mFOLFOX, bevacizumab, and weekly ip PTX, and the other three patients underwent CAPOX, bevacizumab, and weekly ip PTX treatment. The dose of PTX was 20 mg/m [2]. The primary endpoint was the safety of the chemotherapy, and secondary endpoints were response rate, peritoneal cancer index improvement rate, rate of negative peritoneal lavage cytology, progression-free survival, and overall survival. Dose limiting toxicity was not observed, and the adverse events of ip PTX combined with oxaliplatin-based systemic chemotherapy were similar to those described in previous studies using systemic chemotherapy alone [3, 4]. The response rate was 25%, peritoneal cancer index improvement rate was 50%, and cytology in peritoneal lavage turned negative in all the cases. The progression-free survival was 8.8 months (range, 6.8–12 months), and median survival time was 29.3 months [5], which was longer than that observed in previous studies.

**Conclusion:**

Here, we planned the phase II trial of ip paclitaxel combined with conventional chemotherapy for colorectal cancer with peritoneal carcinomatosis: the iPac-02 trial.

**Supplementary Information:**

The online version contains supplementary material available at 10.1007/s00384-023-04434-5.

## Introduction

Colorectal cancer with peritoneal carcinomatosis is associated with poor prognosis. A few reports have evaluated the outcomes of treatment with chemotherapy limited to peritoneal carcinomatosis, and median survival is reported to be 15.2–16.3months in unresectable peritoneal carcinomatosis in recent studies using monoclonal antibodies against VEGF and EGFR [2, 6]. The low response rate to chemotherapy is one reason for the poor prognosis. Recent studies reported a 0–44.9% response rate [7–11], and the development of more effective chemotherapy such as intraperitoneal chemotherapy is desired. Intraperitoneal chemotherapy using 5-fluorouracil, oxaliplatin, Mitomycin C, or irrinotecan has been done for colorectal cancer in combination with surgical resection [12–14]. However, there are no reports demonstrating the efficacy of intraperitoneal chemotherapy for unresectable colorectal cancer.

Intraperitoneal paclitaxel (ip PTX) administration has been reported to be effective in gastric, ovarian, and pancreatic cancer with peritoneal carcinomatosis [15–18]. Unlike other anticancer drugs, paclitaxel is fat-soluble and is absorbed slowly, which contribute to high intraperitoneal concentration and low blood concentrations. Therefore, it is assumed to be effective against intraperitoneal lesions with fewer side effects [19]. Intravenous PTX was inefficient as a chemotherapeutic treatment for colorectal cancer [20]. However, ip PTX has shown efficacy in a rat model of peritoneal carcinomatosis [21].

## Patients and methods

### Patient selection

The first registration inclusion criteria are shown in Table 1, and the exclusion criteria are shown in Table 2. Briefly, patients with peritoneal carcinomatosis only are eligible. The peritoneal carcinomatosis must be present as a measurable lesion. Patients with resectable peritoneal carcinomatosis, microsatellite instability (MSI)-high, contraindication for chemotherapy, and those treated with chemotherapy for peritoneal carcinomatosis are excluded. Peritoneal caricinomatosis is unresectable when it is in a “diffuse metastases to the distant peritoneum” state according to the Japanese guidelines [22]. This corresponds to more than 10 according to peritoneal cancer index (PCI) [23]. Patients with RAS wild type left-sided colorectal cancer can be enrolled. Anti-EGFR antibody contributed to prolonged prognosis in the case of RAS wild-type left-sided cancer [24]. However, bevacizumab is also acceptable for first line of chemotherapy in the Japanese guideline as it might be associated with reduced side effect.

**Table 1:** Inclusion criteria of iPac-02 trial

**Inclusion criteria**
(1) Histologically confirmed initial or recurrent colorectal adenocarcinoma*
(2) The size of peritoneal carcionmatosis confirmed by CT scans is 10 mm or more
(3) Cases without distant metastasis other than peritoneal carcinomatosis
(4) Chemotherapy for peritoneal carcinomatosis has not been performed
(5) Adequate function of important organs (within 14 days before registration) White blood cell ≥ standard lower limit value of JCCLS**, ≤ 12,000/mm^3^ Neutrophil ≥ 1,500/mm^3^ Hemoglobin ≥ 8.0 g/dL Platelet ≥ 100,000/mm^3^ AST ≤ 100U/L, ALT ≤ 100U/L T-Bilirubin ≤ 1.5 times of standard upper limit value of JCCLS** eGFR ≥ 50 mL/min/1.73m2
(6) ECOG performance status: 0–1
(7) The patient survival is expected to be longer than 3 months after registration
(8) Age ≥ 20, < 80 years, at the time of consent
(9) Written consent for participation in the study

*Appendix cancer and anal canal cancer are excluded

**Japanese Committee for Clinical Laboratory Standards

**Table 2:** Exclusion criteria of iPac-02 trial

**Exclusion criteria**
(1) Concurrent double cancers
(2) Cases with a large amount of ascites requiring drainage
(3) The peritoneal carcinomatoses are resectable
(4) Less than 180 days from the end of chemotherapy including oxaliplatin to the appearance of peritoneal carcinomatoses
(5) Contraindicated cases of fluorouracil, levofolinate, capecitabine, oxaliplatin, bevacizumab or paclitaxel
(6) Cases with active infections or unhealed wounds
(7) Pregnancy, breast feeding or intention to become pregnant
(8) Cases participating other clinical trial
(9) Cases with active bleeding, active ulcer lesions and severe gastrointestinal stenosis (but not applicable to cases with stoma)
(10) Complications such as uncontrolled hypertension, severe diarrhea, serious heart disease, or coagulation abnormalities
(11) Cases with brain tumors (including brain metastases)
(12) Cases with arterial thromboembolism
(13) Microsatellite instability (MSI)-high
(14) Judged inappropriate for this trial for other reasons

A total of 38 patients will be enrolled in the study. The number of drop out due to the port system will be very small. The adverse events related to peritoneal access ports, such as catheter infections or obstructions, were observed in 6% of the cases in phase III trial for gastric cancer [18]. However, no patients dropped out because of the port system. There were no adverse events related to peritoneal access port in phase I trial for colorectal cancer [1]. Written informed consent will be obtained from all the patients. the University of Tokyo Ethics Committee approved this study (2021036-11DX). The study was registered Japan Registry of Clinical Trials (jRCT2031220110).

### Endpoints

The response rate is the primary endpoint. Progression-free survival, overall survival, peritoneal cancer index improvement rate, rate of negative peritoneal lavage cytology, safety, and response rate to peritoneal metastases are the secondary endpoints.

The response rate to peritoneal metastases was the response rate measured for peritoneal metastases only. The response rate for peritoneal carcinomatosis is the same as that after the resection of the primary tumor. Progression-free survival is the time from the registration to the time of disease progression or death from any cause. Overall survival is the time from the registration to death from any cause.

### Study design and procedure

**Fig. 1:**
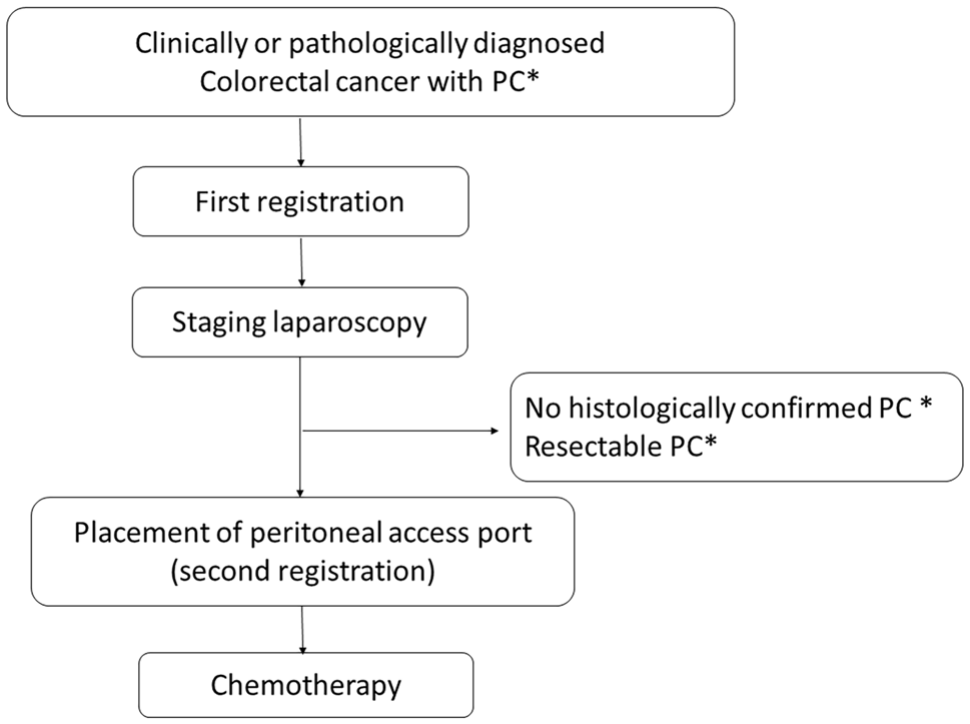
Flow chart of the iPac-02 study. *PC: peritoneal carcinomatosis

This is a multicenter, open-label, single assignment interventional clinical study. This study will be conducted as a physician-initiated clinical trial. The patients undergo staging laparoscopy after the first registration (Fig. 1). When the peritoneal carcinomatosis is pathologically confirmed and is unresectable during laparoscopy, a secondary registration is performed. The peritoneal access port (Bard Peritoneal Titanium port; Becton Dickinson, Covington, GA, USA) is implanted after the secondary registration. The catheter tip of peritoneal access port is placed in pelvis using laparoscopy.

### Assessment

Response rate is the rate of the partial response or complete response evaluated by Response Evaluation Criteria in Solid Tumors (RECIST) version 1.1 [25]. Second-look laparoscopy is performed every 6 months, and PCI improvement and resectability is evaluated. Toxicity was evaluated according to Common Terminology Criteria for Adverse Events (CTCAE) version 5.0 [26]. Progression-free survival and overall survival curves were obtained using the Kaplan–Meier method.

### Chemotherapeutic regimen

**Fig. 2:**
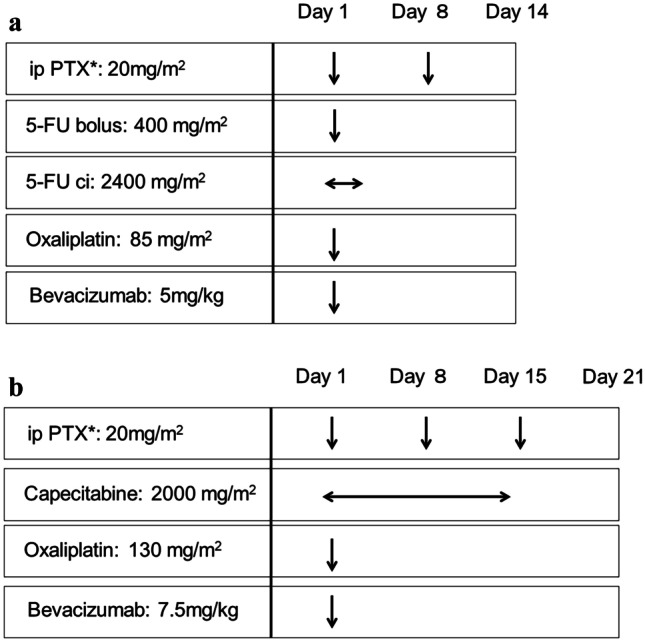
Chemotherapy regimen. The chemotherapy protocol of the (**a**) FOLFOX and (**b**) CapeOX regimens. *IP PTX: intraperitoneal paclitaxel

The FOLFOX-bevacizumab or CAPOX-bevacizumab chemotherapeutic regimens are given concomitantly in the present study. The details of each regimen are described in our previous paper [1]. The schedule is shown in Fig. 2. The FOLFOX regimen is a 14-day cycle that includes ip PTX (20 mg/m^2^, days 1 and 8), bolus 5-FU (400 mg/m^2^, day 1), continuous 5-FU (2400 mg/m^2^, 46 h), calcium levofolinate hydrate (200 mg/m^2^, day 1), oxaliplatin (85 mg/m^2^, day 1), and bevacizumab (5 mg/kg, day 1). The CAPOX regimen is a 21-day cycle that includes ip PTX (20 mg/m^2^, days 1, 8, and 15), capecitabine (2000 mg/m^2^/day, days 1–14), oxaliplatin (130 mg/m^2^, day 1), and bevacizumab (7.5 mg/kg, day 1). Patients can select one of the regimens per course. PTX is dissolved in 500-ml normal saline and administrated intraoperatively using a peritoneal access port after 500-ml normal saline administration.

Chemotherapy administration criteria are shown in Supplementary Table 1. The criteria consist of neutrophils ≥ 1500/mm^3^, platelets ≥ 75,000/mm^3^, peripheral neuropathy ≤ grade 2, and so on. Chemotherapy is interrupted until the criteria are met. The dose reduction criteria and the reduced doses are shown in Supplementary Tables 2 and 3, respectively. Oxaliplatin should be reduced when peripheral neuropathy ≥ grade 2 is observed. Capecitabine should be reduced when grade 2 hand-foot syndrome is observed twice. If the dose reduction criteria are met after a two-step reduction, the drug should be discontinued. Paclitaxel dose reduction is not necessary when hematotoxicity occurs; however, when an adverse event associated with paclitaxel occurred, the dose is reduced to 10 mg/m^2^. The bevacizumab and calcium levofolinate hydrate cannot be reduced. The bevacizumab can be skipped according to the criteria for Supplementary Table 1. In addition, doses can be reduced at the physician’s discretion to ensure safety.

If the adverse event associated with paclitaxel occurred after reduction and continuing chemotherapy was very difficult, the protocol should be discontinued. Treatment is continued until disease progression or until peritoneal carcinomatosis becomes resectable and curative resection can be performed.

### Statistical methods

The study sample size was calculated on the basis of Simon’s two-stage design [27, 28], and the multiplicity was controlled accordingly. The response rate of peritoneal carcionmatosis of colorectal cancer was reported to be 0–44.9% [7–11]. However, these were mainly evaluated pathologically. Very few reports have evaluated response rate of peritoneal carcinomatosis using RECIST. The response rate evaluated by PCI was 28.1–35.6% [7, 9], and the response rate evaluated using RESIST was 0–20.8% [8, 9, 11]. Therefore, we assumed the null hypothesis of ≤ 20%. Although the response rate was 25% in our phase I trial, PCI markedly improved 50% of cases [5]. Therefore, we assumed the expected response rate of ≥ 40% for the primary endpoint, and set one-sided significance level to ≤ 5% and power to ≥ 80%. A total of 38 patients were required. The number of drop out patients was not included because the registration speed is slow and the response can be evaluated even if the treatment discontinued. At the interim analysis, the study will continue to the second stage if at least four 4 patients (29%) among of the first 14 patients respond to the study treatment. At the final analysis if a response is obtained from 12 (32%) out of 38 patients, the null hypothesis will be rejected. The primary analysis will be conducted in the full analysis set composed of all patients who received the study treatment. Response rate and Clopper–Pearson 90% and 95% confidence interval (CI) will be calculated.

For the secondary endpoints, progression-free and overall survival will be analyzed by using the Kaplan–Meier method with 6- and 12-month event-free proportions and their CIs calculated by Greenwood’s formula. In addition, the median survival time and its Brookmeyer–Crowley 95% CI will additionally be calculated. Other secondary endpoints will be summarized by proportions and Clopper–Pearson 95% CIs.

### Future perspective

If the efficacy of ip PTX is observed in this phase II trial, we plan to proceed to randomized controlled phase III trial with interim analysis to compare the efficacy of this regimen in terms of progression-free survival.

### Supplementary Information

Below is the link to the electronic supplementary material.

Supplementary file1 (DOCX 31 KB)

## Data Availability

The datasets generated and/or analyzed during the current study are available from the corresponding author on reasonable request.
