# Supplementary material for: Intraperitoneal paclitaxel combined with FOLFOX/CAPOX plus bevacizumab for colorectal cancer with peritoneal carcinomatosis (the iPac-02 trial): study protocol of a single arm, multicenter, phase 2 study
Source: Int J Colorectal Dis. 2023 Jun 20;38(1):173. doi: 10.1007/s00384-023-04434-5 (PMC10282041; doi:10.1007/s00384-023-04434-5)
Supplement: Supplementary file 1 — Supplementary file1 (DOCX 31 KB) [file 384_2023_4434_MOESM1_ESM.docx]

**supplementary Table 1.** Criteria for chemotherapy administration

|  |
| --- |
| 1. Neutrophil ≥ 1,500/mm^3^ |
| 1. Platelet ≥ 75,000/mm^3^ |
| 1. T-Bilirubin ≤1.5 times of standard upper limit value of JCCLS* |
| 1. AST ≤ 100U/L, ALT ≤ 100U/L |
| 1. Creatinine ≤1.2 times of standard upper limit value of JCCLS* |
| 1. No symptom of active infections |
| 1. Nausea, vomiting, diarrhea, and hand-foot syndrome ≤ Grade 1 |
| 1. Peripheral neuropathy ≤ Grade 2 |
| 1. Hypertension ≤ Grade 2 |
| 1. Proteinuria ≤ 2+ |
| 1. Thrombosis ≤ Grade 2 |
| 1. Bleeding ≤ Grade 1 |
| 1. No lung hemorrhage |
| 1. No unhealed wounds |

*Japanese committee for clinical laboratory standards

Chemotherapy other than bevacizumab can be administered even when criteria (8-14) are not met.

**supplementary Table 2.** Criteria for dose reduction

| FOLFOX regimen | 5-FU | oxaliplatin | paclitaxel |
| --- | --- | --- | --- |
| Neutrophil < 500/mm^3^ | reduction | reduction | No change |
| Platelet < 25,000/mm^3^ | reduction | reduction | No change |
| Febrile neutropenia | reduction | reduction | No change |
| Nausea, diarrhea ≥ Grade 3 | reduction | reduction | No change |
| Peripheral neuropathy ≥ Grade 2 | No change | reduction | No change |
| Adverse event associated with paclitaxel | No change | No change | reduction |
| Adverse events requiring  postponement for more than 15 days | reduction | reduction | No change |

| CAPOX regimen | Capecitabine | oxaliplatin | paclitaxel |
| --- | --- | --- | --- |
| Neutrophil < 500/mm^3^ | reduction | reduction | No change |
| Platelet < 25,000/mm^3^ | reduction | reduction | No change |
| Febrile neutropenia | reduction | reduction | No change |
| Nausea, diarrhea ≥ Grade 3 | reduction | reduction | No change |
| Hand-foot syndrome Grade 2 | 1^st^ No change  2^nd^ reduction | No change | No change |
| Hand-foot syndrome ≥ Grade 3 | reduction | reduction | No change |
| Peripheral neuropathy ≥ Grade 2 | No change | reduction | No change |
| Adverse event associated with paclitaxel | No change | No change | reduction |
| Adverse events requiring  postponement for more than 15 days | reduction | reduction | No change |

**supplementary Table 3.** Reduced dose of chemotherapy

| FOLFOX regimen | Initial dose | Reduction-1 | Reduction-2 |
| --- | --- | --- | --- |
| 5-FU bolus | 400 mg/m^2^ | 0 mg/m^2^ | 0 mg/m^2^ |
| 5-FU continuous | 2,400 mg/m^2^ | 2,000 mg/m^2^ | 1,600 mg/m^2^ |
| oxaliplatin | 85 mg/m^2^ | 65 mg/m^2^ | 50 mg/m^2^ |
| paclitaxel | 20 mg/m^2^ | 10 mg/m^2^ | 0 mg/m^2^ |

| CAPOX regimen | Initial dose | Reduction-1 | Reduction-2 |
| --- | --- | --- | --- |
| oxaliplatin | 130 mg/m^2^ | 100 mg/m^2^ | 85 mg/m^2^ |
| paclitaxel | 20 mg/m^2^ | 10 mg/m^2^ | 0 mg/m^2^ |

Capecitabine

| Body surface area | Reduction-1 | Reduction-2 |
| --- | --- | --- |
| < 1.41 m^2^ | 1,800 mg/m^2^/day | 1,200 mg/m^2^/day |
| 1.41 m^2^ ≤ BSA < 1.51m^2^ | 2,400 mg/m^2^/day | 1,200 mg/m^2^/day |
| 1.51 m^2^ ≤ BSA < 1.81 m^2^ | 2,400 mg/m^2^/day | 1,800 mg/m^2^/day |
| 1.81 m^2^ ≤ BSA < 2.11 m^2^ | 3,000 mg/m^2^/day | 1,800 mg/m^2^/day |
| ≥ 2.11 m^2^ | 3,000 mg/m^2^/day | 2,400 mg/m^2^/day |
